# Carbon dioxide fixation via production of succinic acid from glycerol in engineered *Saccharomyces cerevisiae*

**DOI:** 10.1186/s12934-022-01817-1

**Published:** 2022-05-28

**Authors:** Zahabiya Malubhoy, Frederico Mendonça Bahia, Sophie Claire de Valk, Erik de Hulster, Toni Rendulić, Juan Paulo Ragas Ortiz, Joeline Xiberras, Mathias Klein, Robert Mans, Elke Nevoigt

**Affiliations:** 1 Department of Life Sciences and Chemistry, Jacobs University Bremen gGmbH, Campus Ring 1, 28759 Bremen, Germany; 2 Department of Biotechnology, Delft University of Technology, van der Maasweg 9, 2629 HZ Delft, The Netherlands; 3 Dipartimento Di Biotecnologie E Bioscienze, Università Degli Studi Di Milano-Bicocca, Piazza della Scienza, 4, 67056 Milan, Italy; 4 Present Address: RB/WD - A030, BASF SE, Carl-Bosch-Strasse 38, 67056 Ludwigshafen am Rhein, Germany

**Keywords:** Succinic acid, Glycerol, Yeast, Metabolic engineering, CO_2_ fixing, *Saccharomyces cerevisiae*, Carbon dioxide, Fermentation, Dicarboxylic acids

## Abstract

**Background:**

The microbial production of succinic acid (SA) from renewable carbon sources via the reverse TCA (rTCA) pathway is a process potentially accompanied by net-fixation of carbon dioxide (CO_2_). Among reduced carbon sources, glycerol is particularly attractive since it allows a nearly twofold higher CO_2_-fixation yield compared to sugars. Recently, we described an engineered *Saccharomyces cerevisiae* strain which allowed SA production in synthetic glycerol medium with a maximum yield of 0.23 Cmol Cmol^−1^. The results of that previous study suggested that the glyoxylate cycle considerably contributed to SA accumulation in the respective strain. The current study aimed at improving the flux into the rTCA pathway accompanied by a higher CO_2_-fixation and SA yield.

**Results:**

By changing the design of the expression cassettes for the rTCA pathway, overexpressing *PYC2*, and adding CaCO_3_ to the batch fermentations, an SA yield on glycerol of 0.63 Cmol Cmol^−1^ was achieved (i.e. 47.1% of the theoretical maximum). The modifications in this 2nd-generation SA producer improved the maximum biomass-specific glycerol consumption rate by a factor of nearly four compared to the isogenic baseline strain solely equipped with the dihydroxyacetone (DHA) pathway for glycerol catabolism. The data also suggest that the glyoxylate cycle did not contribute to the SA production in the new strain. Cultivation conditions which directly or indirectly increased the concentration of bicarbonate, led to an accumulation of malate in addition to the predominant product SA (ca. 0.1 Cmol Cmol^−1^ at the time point when SA yield was highest). Off-gas analysis in controlled bioreactors with CO_2_-enriched gas-phase indicated that CO_2_ was fixed during the SA production phase.

**Conclusions:**

The data strongly suggest that a major part of dicarboxylic acids in our 2nd-generation SA-producer was formed via the rTCA pathway enabling a net fixation of CO_2_. The greatly increased capacity of the rTCA pathway obviously allowed successful competition with other pathways for the common precursor pyruvate. The overexpression of *PYC2* and the increased availability of bicarbonate, the co-substrate for the PYC reaction, further strengthened this capacity. The achievements are encouraging to invest in future efforts establishing a process for SA production from (crude) glycerol and CO_2_.

**Supplementary Information:**

The online version contains supplementary material available at 10.1186/s12934-022-01817-1.

## Introduction

The use of renewable resources for the production of chemicals, fuels and materials by microbial cell factories is a central aspect of industrial biotechnology contributing to the vision of a circular economy [1]. The term ‘renewable’ concerns the direct or indirect use of present-day CO_2_ instead of fossil resources. We indirectly use CO_2_ in industrial biotechnology as soon as the feedstocks (e.g. sugars, glycerol or lipids) originate from biomass generated by CO_2_-fixing autotrophic organisms. However, CO_2_ can also be directly used as a substrate in the production of chemicals by heterotrophic microbes provided that a reduced (and energy rich) co-substrate is used and that the target product is more oxidized than this co-substrate (when normalized to the number of carbon atoms). A popular example for such a mixed substrate utilization is the production of organic acids [2]. The latter authors scrutinized the thermodynamic feasibility of such processes for different acids and different (co-) substrates. The study illustrates that the theoretical CO_2_-fixation capacity increases with increasing electron density of the co-substrate [i.e. degree of reduction (d.o.r.) per carbon atom].

Succinic acid (SA) is an organic acid which has been among the top 12 platform chemicals that can be obtained from carbohydrate biomass [3, 4]. Apart from its potential conversion to numerous valuable chemical compounds such as adipic acid, 1,4-butanediol, *γ*-butyrolactone, and tetrahydrofuran, SA can also be utilized as a monomer for the production of bio-based polyesters [5–7]. An attractive example is the biodegradable polyester polybutylene succinate (PBS), which is derived from the polymerization of SA with 1,4-butanediol [8, 9]. For these reasons, several companies (e.g. BASF, Corbion, Myriant and Reverdia) have included SA as a sustainable building block in their research portfolios [8].

High SA titers and productivities have been achieved in industrial fermentations using bacteria [5]. However, a drawback of using bacteria for organic acid production is their low tolerance towards acidity [10]. In contrast, fungi (including yeasts) tolerate low pH values, reducing the need for cultivation broth neutralization [5, 11, 12]. Consequently, the downstream processing is facilitated since the formation of salts such as gypsum is reduced resulting in significant cost reduction when considering the overall process [5, 8]. Thus, fungal organisms are very attractive for the production of organic acids such as SA. Two fungal organisms, i.e. the yeast species *Saccharomyces cerevisiae* and *Pichia kudriavzevii*, have already been exploited for SA production at industrial scale [5]. The yeast *S. cerevisiae* has the great advantage that it is easily accessible for extensive metabolic engineering endeavors. Moreover, its long-term safe use in food and beverage industry made it a popular cell factory in industrial biotechnology.

Among the metabolic pathways leading to SA formation in nature, the fermentative route via the reductive branch of the TCA cycle, also known as reverse TCA (rTCA), has been considered highly attractive for large-scale SA production as it allows the fixation of 1 mol CO_2_ per mole SA produced [5, 8, 12, 13]. The rTCA pathway has been established in the *S. cerevisiae* strains constructed by the company Reverdia and exploited to commercially produce SA [14], Van [15]. The processes relied on sugar- and starch-based feedstocks, i.e. mainly glucose [5]. However, the proportion of SA that can be produced from glucose via the rTCA pathway is restricted by the number of electrons that can be provided per mole of carbon of the substrate. This implies that in addition to SA formation via the net NADH requiring conversion of glucose via the rTCA pathway, SA is also formed via the CO_2_-releasing oxidative TCA pathway, which limits the net CO_2_ fixation potential of the process. This limitation can be circumvented by the use of more reduced substrates which have the potential to be fully converted into SA via the rTCA pathway. For example, glycerol is very attractive for the fixation of CO_2_ via the rTCA pathway as illustrated in the following considerations. The theoretical conversion of glucose (d.o.r. = 24; 6 carbon atoms) into SA (d.o.r. = 14; 4 carbon atoms) corresponds to the following equation which represents the highest possible SA yield on glucose when CO_2_ is used as the only additional carbon source:

1 $${\text{7 C}}_{{\text{6}}} {\text{H}}_{{{\text{12}}}} {\text{O}}_{{\text{6}}}  + {\text{ 6 CO}}_{{\text{2}}}  \to {\text{ 12 C}}_{{\text{4}}} {\text{H}}_{{\text{6}}} {\text{O}}_{{\text{4}}}  + {\text{ 6 H}}_{{\text{2}}} {\text{O }}$$

Although this equation does not take biological processes such as cell growth and maintenance into account, it does indicate that optimized cell factories are able to co-consume 0.5 mol of CO_2_ for each mole of SA formed, with the potential to achieve a process that results in net fixation of CO_2_. In contrast, the higher electron density of glycerol as substrate (d.o.r. = 14; 3 carbon atoms) has the potential to fix 1 mol of CO_2_ for each SA formed in mixed substrate fermentations according to the following equation:

2 $${\text{12C}}_{{\text{3}}} {\text{H}}_{{\text{8}}} {\text{O}}_{{\text{3}}}  + {\text{ 12 CO}}_{{\text{2}}}  \to {\text{ 12 C}}_{{\text{4}}} {\text{H}}_{{\text{6}}} {\text{O}}_{{\text{4}}}  + {\text{ 12 H}}_{{\text{2}}} {\text{O}}$$

**Fig. 1:**
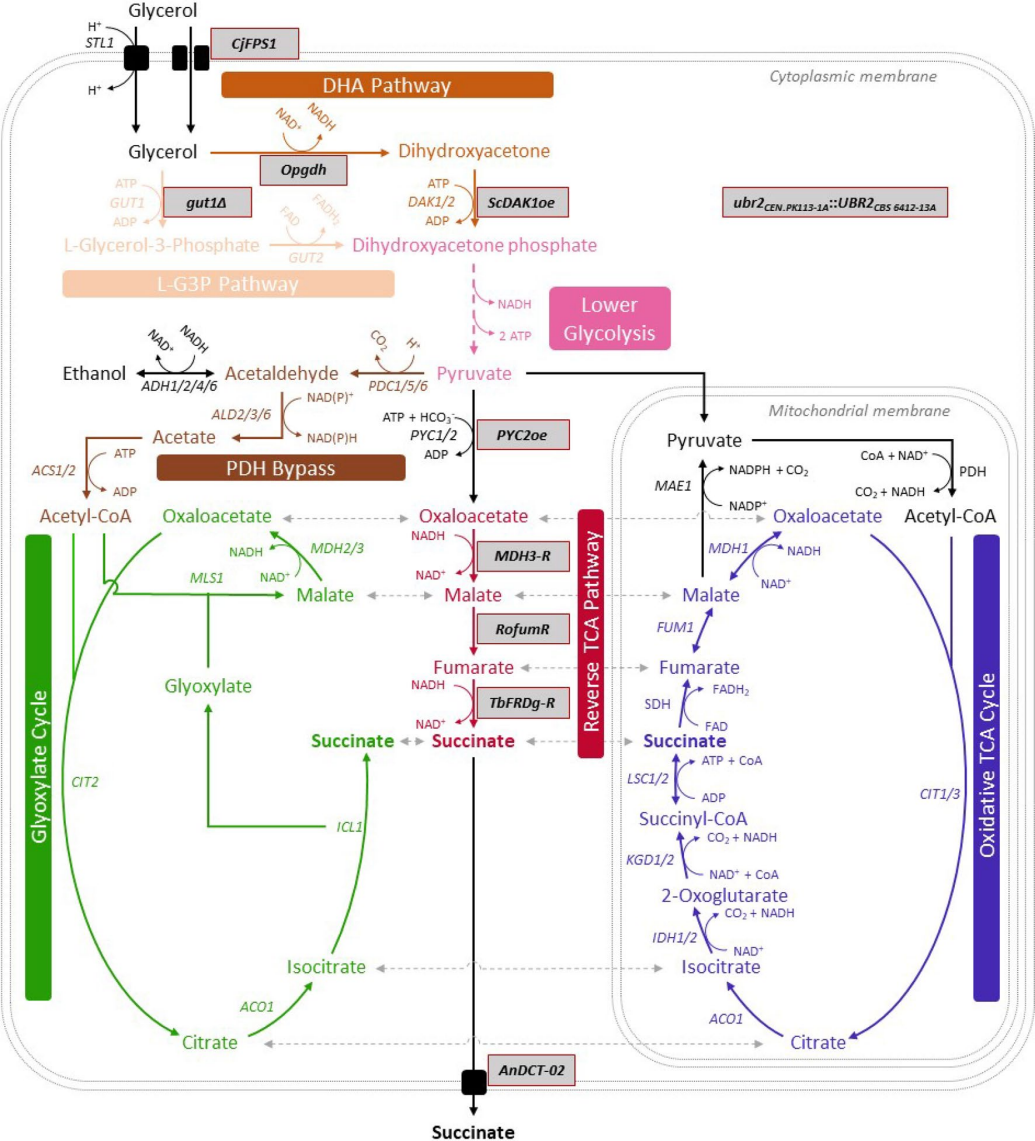
Genetic modifications in our 2nd-generation *S. cerevisiae* strain producing succinic acid (SA) from glycerol*.* All relevant genetic modifications are highlighted in grey. Notably, the 1st-generation strain published by Xiberras et al. [17] did not contain the overexpression of *PYC2.* The peroxisome is not shown. Potential transport of metabolites between cytosol and mitochondria is indicated by dashed arrows in light grey. For more detailed information about known transport mechanisms, the reader is referred to Xiberras et al. [18]. *STL1* glycerol/H^+^ symporter, *DAK1/2* dihydroxyacetone kinase, *PYC1/2* pyruvate carboxylase, *MDH1/2/3* malate dehydrogenase, *FUM1* fumarase, *CIT1/2/3* citrate synthase, *ACO1* aconitase, *ICL1* isocitrate lyase, *MLS1* malate synthase, *MAE1* malic enzyme *ACS1/2* acetyl-coA synthetase, *PDC1/5/6* pyruvate decarboxylase, *ADH1/2/4/6* alcohol dehydrogenase, *ALD2/3/6* aldehyde dehydrogenase, *CjFPS1 Cyberlindnera jadinii FPS1* (aquaglyceroporin*)*, *PYC2oe* *PYC2* overexpression, *MDH3-R* peroxisomal malate dehydrogenase targeted to the cytosol, *RofumR* fumarase from *R. oryzae*, *TbFRDg-R* glycosomal fumarate reductase from *T. brucei* retargeted to the cytosol, *An*DCT-02 dicarboxylic acid transporter from *A. niger,* PDH pyruvate dehydrogenase complex*,* MPC mitochondrial pyruvate carrier, SDH succinate dehydrogenase complex

A crucial prerequisite for the fermentative production of SA from glycerol via the rTCA pathway in *S. cerevisiae* is that the strain is able to efficiently utilize glycerol. Moreover, a redox-factor neutral conversion from substrate to product requires the replacement of the endogenous l-G3P pathway by the so-called DHA pathway (Fig. 1). This strategy for an alternative glycerol catabolism has been previously implemented by Klein et al. [16] for the first time and avoids the direct channeling of electrons from glycerol oxidation into the respiratory chain via the endogenous Gut2 enzyme (FAD-dependent). Instead, the engineered pathway allows transfer of the electrons via a heterologous glycerol dehydrogenase to cytosolic NADH and makes them (together with the electrons stemming from glycolytic NADH) available for the reduction of oxaloacetate to succinate in the rTCA pathway (Fig. 1).

Based on our success in equipping *S. cerevisiae* with an efficient NAD-dependent glycerol catabolism, we recently combined the respective genetic modifications with those required for the establishment of the rTCA pathway for SA production in the cytosol [17]. The genetic modifications for the rTCA pathway (Fig. 1) had been originally described by Van De Graaf et al. [15] and involved the endogenous peroxisomal malate dehydrogenase (*MDH3*), the cytosolic fumarase from *Rhizopus oryzae* (*RofumR*), and the peroxisomal fumarate reductase from *Trypanosoma brucei* (*TbFRDg*). In addition, the dicarboxylic acid transporter from *Aspergillus niger* (*An*DCT-02) was expressed for SA secretion into the medium [17]. Using the resulting strain, a maximum SA titer of ca. 10.7 g L^−1^ and a yield of 0.22 g g^−1^ on glycerol was achieved in ordinary shake flask cultures. However, it became clear that the pyruvate dehydrogenase bypass in combination with the glyoxylate cycle (Fig. 1) significantly contributed to SA production, preventing carbon from entering the envisaged CO_2_-fixing rTCA route. Thus, the initial goal of a redox-balanced, CO_2-_fixing pathway was only partially achieved and about one third of the glycerol consumed was released in the form of CO_2_.

In the current study, we report the construction and evaluation of an optimized 2nd-generation *S. cerevisiae* SA producer with the aim to improve the flux distribution from glycerol to SA via the rTCA pathway, thereby fixing more CO_2_ and resulting in a higher SA yield. Apart from employing new codon-optimized versions of the coding sequences for the rTCA pathway and the dicarboxylic acid transporter for SA export (hereafter referred to as ‘2nd-generation SA module’), the main modifications included promoter/terminator exchanges, alteration of the genomic locations for integrating the expression cassettes of the SA module, and the overexpression of *PYC2*, an isogene encoding pyruvate carboxylase.

## Materials and methods

### Strains, plasmids and maintenance

The plasmids and *S. cerevisiae* strains used in this study are listed in Additional file 2: Table S1 and S4, respectively. Yeast cells were routinely grown on solid YPD medium containing 10 g L^−1^ yeast extract, 20 g L^−1^ peptone, 20 g L^−1^ glucose, and 15 g L^−1^ agar. Agar plates were cultivated in a static incubator at 30 °C. Media were supplemented with phleomycin (20 mg L^−1^), hygromycin B (300 mg L^−1^), or nourseothricin (100 mg L^−1^) for selection purposes when needed. *E. coli* DH5α was used for plasmid construction and isolation, and cells were routinely grown in lysogeny broth (LB) containing 10 g L^−1^ NaCl, 5 g L^−1^ yeast extract, 10 g L^−1^ peptone and adjusted to a pH of 7.5 with 2 M NaOH [19]. For selection and maintenance of plasmid containing cells, 100 mg L^−1^ ampicillin was added. Cultivations were performed on an orbital shaker at 250 rpm and 37 °C and plasmids were isolated by using the GeneJET^™^ Plasmid Miniprep Kit (Thermo Fisher Scientific, Waltham, MA, United States).

### General molecular biology techniques

Preparative PCRs for cloning and sequence determination of integrated expression cassettes were performed using Phusion^®^ High-Fidelity DNA Polymerase (New England BioLabs, Frankfurt am Main, Germany). PCR conditions were adapted to the guidelines of the manufacturer. Restriction enzymes, FastAP alkaline phosphatase and T4 DNA ligase were obtained from Thermo Fisher Scientific and used according to the manufacturer’s instructions. PCR products were purified by using the GeneJET^™^ PCR Purification Kit (Thermo Fisher Scientific) and DNA fragments obtained after restriction were excised and purified using the QIAquick Gel Purification Kit (Qiagen, Hilden, Germany). Transformation of *S. cerevisiae* with plasmids as well as linear expression cassettes for genomic integration was performed according to the lithium acetate method described by [20].

### Construction of expression cassettes for genomic integration

The plasmids used for construction of the expression cassettes are listed in Additional file 2: Table S1. The use of *MDH3* encoding peroxisomal malate dehydrogenase [21] as a part of the rTCA pathway has been described by Yan et al. [22]. *Rhizopus oryzae fumR* (*RofumR*) has been described by Song et al. [23] (GenBank accession number X78576) and glycosomal *FRDg* from *Trypanosoma brucei* (*TbFRDg*) by Besteiro et al. [24] (GenBank accession number AF457132). The dicarboxylic acid transporter DCT-02 from *Aspergillus niger* (*An*DCT-02) has been described by Jansen et al. [14]. The amino acid sequence can be retrieved under the GenBank accession number CAK32633.1 and has 100% identity with sequences designated as a malic acid transport protein from *A. awamori* (accession number GCB24846.1) and a voltage-dependent anion channel from *A.* *welwitschiae* (accession number RDH28203.1). Codon-optimized coding sequences for *RofumR*, *TbFRDg*, and *AnDCT-02* were obtained from Thermo Fisher Scientific and GenScript (Piscataway, USA), respectively (Additional file 2: Table S1). The respective DNA sequences are available in the supplementary material (Additional file 2: Table S6). Mdh3 and Frd were targeted to the cytosol by omitting the last 9 bp during PCR-amplification (see reverse primers in Additional file 2: Table S2) leading to the removal of the peroxisomal targeting signal (SKL) from the protein (coding sequences named *MDH3-R* and *TbFRDg-R* accordingly). The cassettes for *S. cerevisiae MDH3-R* (*ScMDH3-R*) under the control of the *JEN1* promoter and the *IDP1* terminator, *RofumR* under the control of the *HOR7* promoter and the *DIT1* terminator, *TbFRDg-R* under the control of *FBA1* promoter and *ADH1* terminator and for *AnDCT-02* under control of the *COX7* promoter and the *CYC1* terminator, were assembled in pUC18 using Gibson isothermal assembly [25]. All primers used for the amplification of the respective promoters, coding sequences and terminators for Gibson assembly are listed in Additional file 2: Table S2. The coding sequences for *RofumR*, *TbFRDg-R* and *AnDCT-02* were amplified from plasmids pMA-T-*RofumR*, pOK-RQ-*TbFRDg-R* and pUC18-*AnDCT-02* w/o STOP, respectively (Additional file 2: Table S1). The coding sequence for *ScMDH3-R* and all promoters and terminators were amplified from genomic DNA isolated from the *S. cerevisiae* strain CEN.PK113-7D *UBR2*_*JL1*_* GUT1*_*JL1*_ [26]. One-step isothermal DNA assembly reactions were prepared as described by [25] and contained 15 μL of the reagent enzyme mix, 0.05 pmol of PCR-amplified pUC18 vector (see Additional file 2: Table S2 for the used primers) and threefold excess of the inserts (promoter, coding sequence and the terminator (each 0.15 pmol) in a final volume of 20 μL. Reaction mixtures were incubated at 50 °C for 1 h. *E. coli* DH5α cells were transformed with 5 μL of the reaction mixture and the resulting vectors named pUC18-*P*_*JEN1*_*-MDH3-R*, pUC18-P_*HOR7*_-*RofumR*, pUC18-P_*FBA1*_-*TbFRDg-R* and pUC18-P_*COX7*_-*AnDCT-02* (Additional file 2: Table S1).

### Plasmids for CRISPR–Cas9 mediated genome editing in *S. cerevisiae*

For CRISPR–Cas9 mediated genome editing, both Cas9 and the gRNA targeting the endonuclease to the locus of integration were expressed from plasmids (Additional file 2: Table S1). The plasmid p414-*TEF1p-Cas9-CYC1t-hphMX* was constructed in analogy to p414-*TEF1p-Cas9-CYC1t-natMX* [16]. The plasmid p414-*TEF1p-Cas9-CYC1t* was used as a starting point and was a gift from George Church (Addgene plasmids #43802). The auxotrophic *TRP1* marker was replaced by an expression cassette conferring resistance to hygromycin B (in p414-*TEF1p-Cas9-CYC1t*) by in vivo homologous recombination in *S. cerevisiae* CEN.PK113-1A. The marker cassette was amplified from pAG32 [27], using primers 445 and 446 (Additional file 2: Table S2). The primers contained 5' terminal sequences of 60 bp homologous to regions upstream and downstream of the *TRP1* marker cassettes in p414-*TEF1p-Cas9-CYC1t*. The vector was linearized within the *TRP1* coding sequence using *Mun*I. CEN.PK113-1A was then co-transformed with the linearized vector and the PCR-amplified resistance marker for assembly by in vivo homologous recombination and the resulting vector named p414-*TEF1p-Cas9-CYC1t-hphMX*.

### *S. cerevisiae* strain construction

#### General strategies for genomic integrations via CRISPR–Cas9

For genomic integrations via CRISPR–Cas9 the long terminal repeats *YGLCτ3* and *YPRCτ3* on chromosomes VII and XVI, respectively [28], and position XI-3 [29] were used. In order to target Cas9 to the aforementioned genomic locations, the vectors p426-*SNR52p-gRNA.YGLCt3-SUP4t-hphMX*, p426-*SNR52p-gRNA.YPRCt3-SUP4t-hphMX* and pCfB3045 that carry expression cassettes for the respective gRNAs were used (Additional file 2: Table S1). According to the employed resistance marker for gRNA expression, Cas9 was expressed using either plasmid p414-*TEF1p-Cas9-CYC1t-nat1* or p414-*TEF1p-Cas9-CYC1t-hphMX* (Additional file 2: Table S1). DNA fragments for assembly and integration (either entire expression cassettes or parts thereof) were PCR-amplified from the vectors listed in Additional file 2: Table S1 or from genomic DNA isolated from strain CEN.PK113-1A *UBR2*_*JL1*_* GUT1*_*JL1*_ [26]. The used primers (Additional file 2: Table S3) contained 5'-extensions generating 40–60 bp sequences homologous to regions directly upstream and downstream of the inserted double strand break at the integration site or to the respective adjacent fragment (in case several cassettes were assembled at the same locus). Co-transformation of the *S. cerevisiae* strain expressing the Cas9 endonuclease with equimolar amounts of the expression cassettes and the respective vector for gRNA expression resulted in assembly and integration of all expression cassettes at the target locus. Positive transformants were selected on YPD agar containing both nourseothricin and hygromycin B. Both vectors were subsequently removed from the resulting clone by serial transfers in YPD medium lacking the respective antibiotics yielding the desired strain. Subsequently, all integrated expression cassettes were sequenced.

#### Gene deletions

The deletions of *GUT1* and *ICL1* were obtained using disruption cassettes consisting of the phleomycin resistance marker (*ble*) and nourseothricin resistance marker (*natMX*), respectively. The disruption cassettes were amplified from plasmids pUG66 and pUG74 (Additional file 2: Table S1) using primer pairs 111/476 and 1189/1190, respectively (Additional file 2: Table S3). The primers used for amplification contained at their 5' terminal end a 60 bp sequence complementary to the region immediately upstream or downstream of the start or stop codon of the gene to be deleted. After PCR amplification and purification, the disruption cassettes were used for transformation of the respective *S. cerevisiae* strains.

#### Construction of strain UBR2_CBS_-DHA (2)

The strain CEN.PK113-1A *UBR2*_*CBS*_* GUT1*_*JL1*_, which had been previously constructed in our lab by replacement of the endogenous *UBR2* and *GUT1* alleles by those from CBS 6412-13A and CEN.PK113-7D *JL1*, served as a starting point in the current study. The allele replacements were achieved as described in Ho et al. [26]. First, the modified version of the DHA module (consisting of the three expression cassettes for *Opgdh, DAK1* and *CjFPS1*) was assembled and integrated in this strain using CRISPR–Cas9. For this purpose, five PCR fragments were generated (in order of integration: the *Opgdh* expression cassette, the *ADH2* promoter, a fragment consisting of the *DAK1* coding sequence and the *TPS1* terminator, the *TDH3* promoter, and a fragment consisting of the *CjFPS1* coding sequence and the *RPL15A* terminator). The primers and DNA templates used for amplification are listed in Additional file 2: Table S2. The purified PCR products were then assembled and integrated at the *YGLCτ3* locus of strain CEN.PK113-1A *UBR2*_*CBS*_* GUT1*_*JL1*_ via CRISPR–Cas9 as described above. Subsequently, the *GUT1* allele was deleted using the phleomycin disruption cassette. The resulting strain was named UBR2_CBS_-DHA (2).

#### Construction of the 2nd-generation SA producer UBR2_CBS_-DHA-SA-AnDCT02 (2)

The new baseline strain UBR2_CBS_-DHA (2) was used to generate the 2nd-generation SA producer by integrating expression cassettes for the rTCA pathway and the dicarboxylic acid transporter *An*DCT-02. Apart from obtaining a stronger flux through the rTCA pathway, another motivation for the effort to modify the SA module was to generate an SA-producing strain which is independent of the DNA constructs kindly provided by DSM for the study of Xiberras et al. [17] providing future academic research more freedom to operate. The expression cassettes for *ScMDH3-R*, *RofumR*, *TbFRDg-R* and *AnDCT-02* were amplified from the previously assembled plasmids pUC18-*P*_*JEN1*_*-MDH3-R*, pUC18-P_*HOR7*_-*RofumR*, pUC18-P_*FBA1*_-*TbFRDg-R* and pUC18-P_*COX7*_-*AnDCT-02* (Additional file 2: Table S1) using the primer pairs listed in Additional file 2: Table S3. These cassettes were subsequently assembled and integrated at the *YPRCτ3* locus of strain UBR2_CBS_-DHA by employing the CRISPR–Cas9 system as described above to yield the reconstructed strain *UBR2*_*CB*S_-DHA-SA-*An*DCT-02.

#### Strain UBR2_CBS_-DHA-SA-AnDCT-02 (2)-PYC2oe

The cassette for overexpression of endogenous *PYC2* (under the control of the *HOR7* promoter and the *TPS1* terminator) was assembled and integrated using CRISPR–Cas9 at position XI-3 in the reconstructed strain *UBR2*_*CB*S_-DHA-SA-*An*DCT-02 (2). Promoter, coding sequence and terminator were amplified from genomic DNA of strain CEN.PK113-1A *UBR2*_*JL1*_* GUT1*_*JL1*_ using the primers listed in Additional file 2: Table S3. CRISPR–Cas9 mediated assembly and integration resulted in strain *UBR2*_*CBS*_-DHA-SA-*An*DCT-02 (2)-*PYC2oe*.

#### Isolation of genomic DNA from *S. cerevisiae* transformants and diagnostic PCR

Correct integration of all expression and disruption cassettes was verified by diagnostic PCR using OneTaq Quick-load DNA polymerase and buffer according to the manufacturer’s guidelines (New England Biolabs). Genomic DNA was isolated according to a modified protocol from Hoffman and Winston [30]. Approximately 50 mg of cells were suspended in 200 µL of TE buffer (10 mM Tris, 1 mM EDTA, pH 8.0). Subsequently, 300 mg of acid-washed glass beads (diameter of 0.425–0.6 mm) and 200 µL of phenol:chloroform:isoamyl alcohol (25:24:1) were added. The tubes were vortexed at maximum speed for 2 min and centrifuged at 15,700*g* for 10 min. The aqueous phase (1 µL) was used as template in 25 µL PCR reactions. PCR primers were designed to bind upstream and downstream of the genomic integration sites as well as within the integrated expression/deletion cassette. For analyzing integrations of multiple expression cassettes, additional primers were designed to produce amplicons covering the junctions between the individual integrated expression cassettes.

### Media and cultivation conditions for the production of SA from glycerol

All pre- and intermediate cultures were cultured in synthetic medium-containing 20 g L^−1^ glucose and ammonium sulfate as the carbon and nitrogen source, respectively. All experiments for assessing SA production in shake flask batch cultivation were performed in synthetic medium-containing 60 mL L^−1^ (75.6 g L^−1^) glycerol as the sole carbon source with urea as the nitrogen source. The synthetic medium was prepared according to Verduyn et al. [31] containing 3 g L^−1^ KH_2_PO_4_, 0.5 g L^−1^ MgSO_4_·7H_2_O, 15 mg L^−1^ EDTA, 4.5 mg L^−1^ ZnSO_4_·7H_2_O, 0.84 mg L^−1^ MnCl_2_·2H_2_O, 0.3 mg L^−1^ CoCl_2_·6H_2_O, 0.3 mg L^−1^ CuSO_4_·5H_2_O, 0.4 mg L^−1^ NaMoO_4_·2H_2_O, 4.5 mg L^−1^ CaCl_2_·2H_2_O, 3 mg L^−1^ FeSO_4_·7H_2_O, 1 mg L^−1^ H_3_BO_3_, and 0.1 mg L^−1^ KI. After heat sterilization of the medium, filter sterilized vitamins were added. Final vitamin concentrations were: 0.05 mg L^−1^ d-( +)-biotin, 1 mg L^−1^ d-pantothenic acid hemi-calcium salt, 1 mg L^−1^ nicotinic acid, 25 mg L^−1^ myo-inositol, 1 mg L^−1^ thiamine chloride hydrochloride, 1 mg L^−1^ pyridoxine hydrochloride, and 0.2 mg L^−1^ 4-aminobenzoic acid. In case urea was used as the nitrogen source (in main culture media), an appropriate aliquot of a stock solution was added after autoclaving to obtain a final concentration of 2.8 g L^−1^ while in all other cultures 5 g L^−1^ ammonium sulfate was added (in preculture media) before heat sterilization. The pH of the synthetic glucose medium was adjusted to 6.5 with 4 M KOH, while that of synthetic glycerol medium was adjusted to 4.0 with 2 M H_3_PO_4_. For pre-cultivation, cells from a single colony were used to inoculate 3 mL of the synthetic glucose medium in a 10 mL glass tube and incubated at orbital shaking of 200 rpm and 30 °C overnight. The pre-culture was used to inoculate 10 mL of the same medium in a 100 mL Erlenmeyer flask (closed with a metal cap) adjusting an OD_600_ of 0.2. This culture, hereafter referred to as intermediate culture, was cultivated at the same conditions for 24 h. The appropriate culture volume from the intermediate culture (in order to later adjust an OD_600_ of 0.2 in 100 mL of synthetic glycerol medium) was centrifuged at 800 g for 5 min and the supernatant discarded. The cell pellet was then washed once by re-suspending the cells in synthetic glycerol medium. The cell suspension was centrifuged again and re-suspended in 100 mL of the same medium in a 500 mL Erlenmeyer flask (closed with a cotton plug), adjusting to a final OD_600_ of 0.2. The main cultures were incubated at orbital shaking of 200 rpm and 30 °C and samples for OD_600_ determination and HPLC analysis were taken at regular time intervals.

For those cultures which were supplemented with CaCO_3_, 3 g of CaCO_3_ were transferred to a 500 mL shake flask and autoclaved. The synthetic glycerol medium which was used for these cultures had its pH adjusted to 6.0 with 4 M KOH. Main cultures (100 mL) with an initial OD_600_ of 0.2 were prepared as described above and subsequently the entire cell suspensions were added to the shake flasks containing the CaCO_3_ under sterile conditions. For OD_600_ measurements, samples were diluted in 0.2 M HCl ensuring complete dissolving of the suspended CaCO_3_.

### Metabolite analysis by HPLC

Samples of culture supernatants (1 mL) were first filtered through 0.2 mm Minisart RC membrane filters (Sartorius, Göttingen, Germany) and if required stored at −20 °C until analysis. The concentrations of SA, glycerol and ethanol in culture media were determined using a Waters HPLC system (Eschborn, Germany) consisting of a binary pump system (Waters 1525), injector system (Waters 2707), the Waters column heater module WAT038040, a refractive index (RI) detector (Waters 2414) and a dual wavelength absorbance detector (Waters 2487). The samples were loaded on an Aminex HPX-87H cation exchange column (Bio-Rad, München, Germany) coupled to a Micro-guard^R^ column (Bio-Rad) and eluted with 5 mM H_2_SO_4_ as the mobile phase at a flow rate of 0.6 mL min^−1^ and a column temperature of 45 °C. Volumes of 20 µL of sample were used for injection. SA and malate were detected using the dual wavelength absorbance detector (Waters 2487) while ethanol and glycerol were analyzed with the RI detector (Waters 2414). The retention time for MA was 9.7 min, for SA 12.0 min, for glycerol 13.5 min and for ethanol 22.7 min. Data were processed and analyzed using the Breeze 2 software (Waters).

### Bioreactor experiments

For each bioreactor experiment cells from a single colony were used to inoculate 100 mL synthetic medium containing 75.6 g L^−1^ glycerol (prepared as described in “Media and cultivation conditions for the production of SA from glycerol” but instead of urea, ammonium sulfate was used as nitrogen source) in 500 mL round-bottom shake flasks and incubated in an Innova 44 incubator (New Brunswick Scientific, Edison, NJ, United States) set at 30 °C and 200 rpm overnight. Cells growing exponentially in fresh medium were then used to inoculate the bioreactors at an initial OD of 1.0 (~ 0.7 g_CDW_ L^−1^). Aerobic batch cultures were grown in 2-L bioreactors (Applikon, Delft, Netherlands) at a starting working volume of 1 L synthetic glycerol medium with ammonium sulfate (5 g L^−1^) as nitrogen source. In order to sustain higher biomass concentrations, the vitamin and trace elements concentrations were increased (twofold) and additional biotin was added separately to reach a final medium concentration of 1 mg L^−1^ as described by Wahl et al. [32]. The pH of the culture was maintained at 5.0 via automated addition of 2 M KOH and the temperature was maintained at 30 °C. The inflowing gas was an in-line mix of pressurized air and pure CO_2_ (> 99.7% purity, Linde Gas Benelux, Schiedam, Netherlands) at a combined flowrate of 500 mL min^−1^ controlled with mass flow controllers (Brooks, Hatfield, PA, United States). The resulting gas mixture was aimed to contain 10% CO_2_ and 19% oxygen and were continuously measured using an NGA 2000 analyzer (Rosemount Analytical, Orrville, OH). The bioreactor was stirred at 800 rpm. Samples for OD_660_ and dry weight determination and HPLC analysis were taken at regular time intervals. Extracellular metabolite concentrations, with the exception of malate, were determined on an Agilent 1260 HPLC, equipped with a Bio-Rad HPX-87H column. Detection was performed by means of an Agilent refractive index detector and an Agilent 1260 VWD detector. Concentrations of malate were determined using a Bio-Rad HPX-87H 300 column (7.8 mm). The column was eluted with phosphoric acid (1.5 mM, 0.6 mL min^−1^. The detection was performed with a refractometer (Waters 2414 and a UV detector (210 nm; Waters 484. For dry weight measurements, 5 mL of culture were filtered over a pre-weighed nitrocellulose filter with pore size 0.45 μm. Subsequently, these filters were washed with water, dried for 20 min at 360 W in a microwave oven and weighted again.

## Results

### Changing the genetic constitution of the baseline strain UBR2_CBS_-DHA

The 1st-generation of the *S. cerevisiae* strain UBR2_CBS_-DHA-SA-AnDCT-02 engineered for SA production from glycerol reported by Xiberras et al. [17] was constructed based on a DHA pathway derivative of the strain CEN.PK113-1A published by Klein et al. [16]. The respective baseline strain UBR2_CBS_-DHA exhibited the highest growth rate in synthetic glycerol medium at the time (0.26 h^−1^) and carried the so-called “DHA pathway module II” according to Klein et al. [16]. This module consisted of four expression cassettes: one cassette for the heterologous expression of the NAD-dependent glycerol dehydrogenase from *Ogataea parapolymorpha* (*Opgdh*) and two different cassettes for the overexpression of endogenous dihydroxyacetone kinase (*DAK1*). Moreover, the module contained an expression cassette for an aquaglyceroporin from *Cyberlindnera jadinii* (*CjFPS1*) since we assumed that facilitated glycerol uptake might benefit glycerol consumption and growth in the engineered strain. This had been previously shown in the genetic background of the natural isolate CBS 6412-13A [33]. To abolish the carbon flux via the endogenous L-G3P pathway, the entire module had been integrated in the *GUT1* locus, thereby eliminating glycerol kinase activity (the first reaction of the aforementioned pathway). The strain also contained the replacement of the endogenous *UBR2* allele by that from the *S. cerevisiae* strain CBS 6412-13A, which is naturally able to grow moderately in synthetic glycerol medium [33, 34]. This modification proved to be crucial for the improved growth phenotype of CEN.PK strains on glycerol [35].

The previously constructed strain UBR2_CBS_-DHA contains two expression cassettes for the overexpression of *DAK1* [16]. As this genetic constitution bears a risk for recombination events, a new strain referred to as UBR2_CBS_-DHA (2) with only one cassette for *DAK1* expression was constructed in the current study (Additional file 1: Figure S1). The *DAK1* coding sequence was placed under the control of the *ADH2* promoter, which according to Ho et al. [36] leads to a remarkably higher expression on glycerol compared to the previously employed *ACT1* and *TDH3* promoters. The latter authors applied a reporter gene to determine promoter strength in synthetic glycerol medium and confirmed their findings by demonstrating the remarkable impact of the promoter used for *DAK1* overexpression on the strains’ growth on glycerol. In addition to aforementioned modification, the promoter used to control the expression of *CjFPS1* in the strain UBR2_CBS_-DHA (2) was changed from *PGK1* to *TDH3* since the latter was assumed to be stronger according to the results obtained by Ho et al. [36]. The modified DHA pathway module, now consisting of three expression cassettes, was integrated at the *YGLCτ3* locus on chromosome VII [28]. This genomic integration site has been reported by the authors to result in intermediate to high expression levels of reporter expression cassettes. Finally, the endogenous *GUT1* gene was deleted using the phleomycin resistance marker. The newly constructed ‘DHA pathway derivative’ UBR2_CBS_-DHA (2) showed a similar growth rate and glycerol consumption in shake flask cultivations (Additional file 1: Figure S2) compared to the strain UBR2_CBS_-DHA of Xiberras et al. [17].

### Construction of a 2nd-generation of the strain UBR2_CBS_-DHA-SA-AnDCT-02 with and without PYC2 overexpression

To obtain an optimized strain producing SA from glycerol, the next step was the implementation of a 2nd-generation SA module in the strain UBR2_CBS_-DHA (2). Compared to the study of Xiberras et al. [17], we changed several aspects of how the respective expression cassettes were designed (Additional file 2: Table S5). First, the coding sequences were re-synthesized (*RofumR, TbFRDgR* and *AnDCT-02*) using a different codon optimization algorithm as described in Material and Methods. In addition, promoters and/or terminators of all expression cassettes were changed (Additional file 2: Table S5). In this regard, we considered both the strength of the promoters as well as the use of promoter/terminator combinations preventing homologous recombination that could result in a loss of expression cassettes during integration. All expression cassettes required for the re-designed SA module were integrated at the *YPRCτ3* locus on chromosome XVI. This locus has been reported to allow high expression levels of integrated genes [28].

Previous studies aiming at SA production via the rTCA pathway in *S. cerevisiae* using glucose as the carbon source overexpressed *PYC2* encoding pyruvate carboxylase (Van [15, 22]). Initially, we did not overexpress *PYC2* since previous studies had reported that expression levels of the native pyruvate carboxylases on respiratory carbon sources were already high [37–40]. Moreover, the overexpression of *PYC2* was accompanied by a drop in SA production in our 1st-generation SA producer (unpublished data). In the current study, we wanted to test whether this modification affects SA production from glycerol in our 2nd-generation strain. Thus, we equipped the reconstructed strain UBR2_CBS_-DHA-SA-AnDCT-02 (2) with a cassette for *PYC2* overexpression*.* The cassette was integrated in chromosome XI (locus XI-3), according to Jessop-Fabre et al. [29] as described in the Materials and Methods section, and the strain referred to here as UBR2_CBS_-DHA-SA-AnDCT-02 (2)-PYC2oe.

### The SA production in shake flask cultivations was remarkably improved in the 2nd-generation SA producer and PYC2 overexpression further increased the performance

**Fig. 2:**
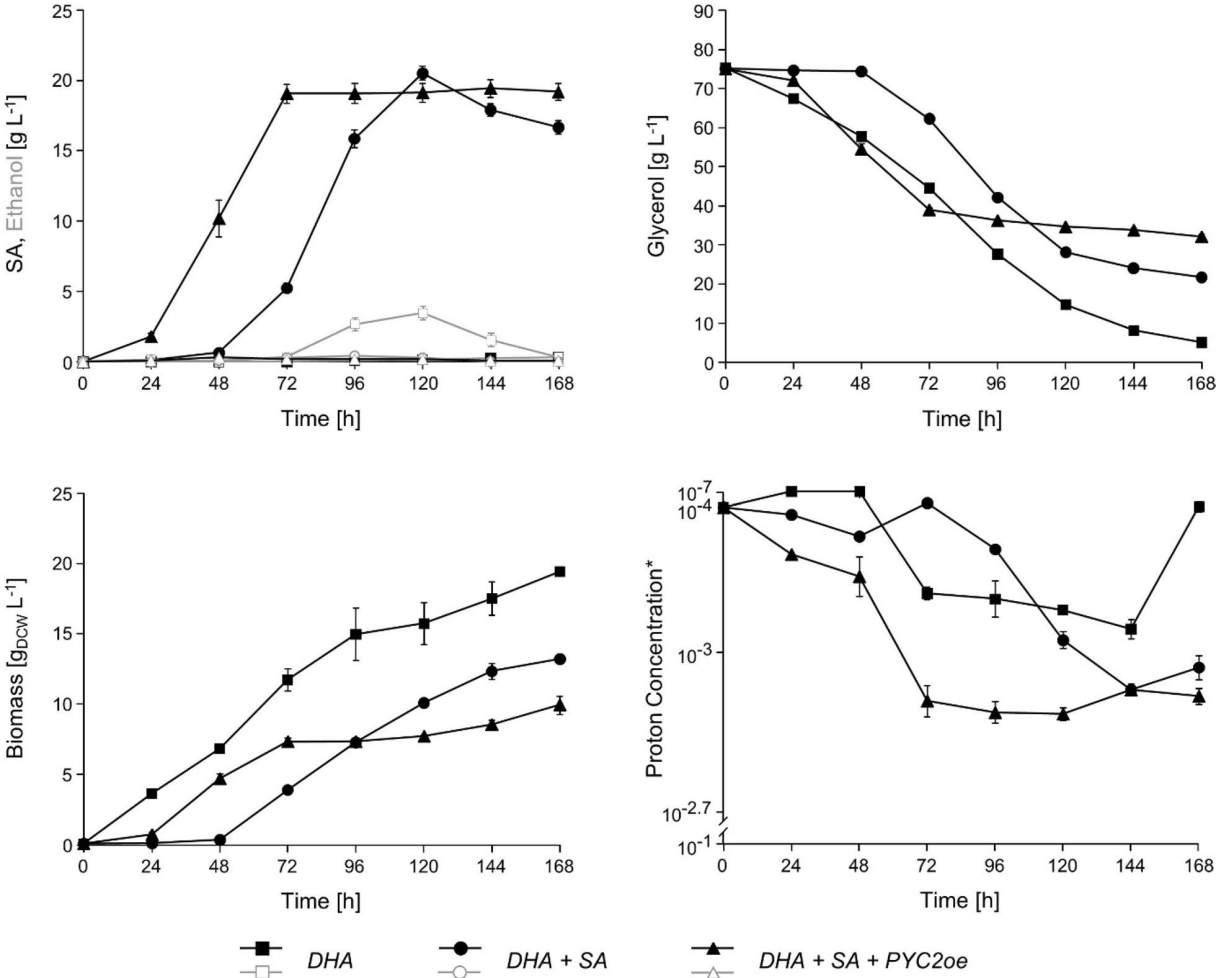
Performance of the 2nd-generation *S. cerevisiae* SA producers in ordinary shake flask cultivations. The new DHA pathway (baseline) strain was used as a control. The cultivations were performed in 500 mL shake flasks filled with 100 mL synthetic glycerol medium at an initial pH of 4 using urea as the nitrogen source. HPLC analysis was used to determine the concentrations of glycerol, SA and ethanol in the culture supernatant. Biomass accumulation was measured by optical density at 600 nm (OD_600_). Mean values and standard deviations were determined from three biological replicates. Strain abbreviations: *DHA* UBR2_CBS_-DHA(2), *DHA* + *SA* UBR2_CBS_-DHA-SA-AnDCT-02 (2), *DHA* + *SA* + *PYCoe* UBR2_CBS_-DHA-SA-AnDCT-02 (2)-PYC2oe.*Instead of pH, the proton concentration is shown, in order to better illustrate the strong decrease throughout the cultivation of the best SA producer

The 2nd-generation SA producer UBR2_CBS_-DHA-SA-AnDCT02 (2) and the isogenic derivative overexpressing *PYC2* (UBR2_CBS_-DHA-SA-AnDCT02 (2)*-*PYC2oe) were first tested in synthetic glycerol medium in batch shake flask cultures under exactly the same conditions as previously described [17]. Both strains showed a maximum accumulation of up to ~ 20 g L^−1^ of SA in the cultivation medium (Fig. 2). The maximum SA yields for UBR2_CBS_-DHA-SA-AnDCT-02 (2) and its *PYC2*-overexpressing derivative were 0.50 ± 0.01 Cmol Cmol^−1^ (0.48 g_SA_ g_glycerol_^−1^) and 0.55 ± 0.03 Cmol Cmol^−1^ (0.53 g_SA_ g_glycerol_^−1^), respectively. These values obtained in shake flask experiments are remarkably higher (more than twofold) compared to those of the 1st-generation SA producing strain UBR2_CBS_-DHA-SA-AnDCT-02 previously published by Xiberras et al. [17]. Moreover, it became apparent that both new strains had a shorter lag phase and produced SA earlier than the previously published 1st-generation SA producer. While the strain constructed by Xiberras et al. [17] reached the maximum SA titer not before 168 h of cultivation, the corresponding new strain (i.e. UBR2_CBS_-DHA-SA-AnDCT-02 (2) without *PYC2* overexpression) reached this maximum already after 120 h. This effect was significantly more pronounced in the new strain overexpressing *PYC2* as it reached the maximum SA titer already after 72 h (Fig. 2).

A closer look at the dataset including biomass formation led us to the hypothesis that the efficient flux into the rTCA pathway has led to an increased carbon flux through glycolysis. We calculated the biomass-specific glycerol consumption rates for each strain throughout the respective cultivation as a proxy to estimate their glycolytic flux rates. As expected, the rates significantly changed over time and the slopes of the changes depended on the strain (Additional file 1: Figure S3). However, it became obvious that the maximum biomass-specific glycerol consumption rate was remarkably higher in the strain UBR2_CBS_-DHA-SA-AnDCT-02 (2) compared to the baseline strain UBR2_CBS_-DHA (2). The overexpression of *PYC2* resulted in an even further increase of this value reaching a level that was nearly four times higher compared to the strain UBR2_CBS_-DHA (2).

Compared to the baseline strain UBR2_CBS_-DHA (2), both new strains, i.e. UBR2_CBS_-DHA-SA-AnDCT-02 (2) with and without *PYC2* overexpression, only produced negligible amounts of ethanol (Fig. 2). In this aspect, the new results confirm the previously reported successful competition of the rTCA route over alcoholic fermentation for pyruvate and NADH [17]. The results presented in Fig. 2 also show that biomass formation, glycerol consumption, and SA production ceased abruptly in the best SA producer (strain UBR2_CBS_-DHA-SA-AnDCT-02 (2)-PYC2oe) when only about 50% of the supplied glycerol had been consumed. Notably, the medium pH had dropped below 3.0 at this stage. This low pH was reached much later in the cultivation using the 2nd-generation SA producer without the *PYC2* overexpression (Fig. 2).

### The glyoxylate cycle does not seem to contribute to SA production in the 2nd-generation SA producers

SA production in our previously published strain UBR2_CBS_-DHA-SA-AnDCT-02 was obviously highly dependent on carbon flux via the PDH bypass and a functional glyoxylate cycle. In fact, the deletion of *ICL1* encoding isocitrate lyase, a key enzyme of the latter pathway, remarkably reduced SA production in this strain [17]. To enable an SA production process which leads to net consumption of CO_2_, it is essential that a significant portion of SA is formed via the CO_2_-fixing rTCA pathway. Thus, any involvement of the glyoxylate cycle in SA production is counterproductive. Therefore, it was of interest to test whether the deletion of *ICL1* had a similar negative impact on the SA production of our 2nd-generation SA producers both carrying the optimized SA module. Similar to the study of Xiberras et al. [17] the possible impact of the glyoxylate cycle was tested by generating *icl1* deletion mutants of the strains UBR2_CBS_-DHA-SA-AnDCT-02 (2) and UBR2_CBS_-DHA-SA-AnDCT-02 (2)-PYC2oe. Interestingly, the deletion of *ICL1* neither resulted in decreased SA production nor an onset of ethanol formation as had been observed by Xiberras et al. [17]. These results obtained with the 2nd-generation strains indicate that the optimized rTCA pathway strongly pulls carbon (and reducing equivalents formed by glycerol dehydrogenase and glycolytic glyceraldehyde 3-phosphate dehydrogenase; Fig. 1) towards fermentative SA production via the envisaged redox-neutral route.

### Addition of CaCO_3_ to shake flask cultures significantly increased the titer and yield of dicarboxylic acids produced

**Fig. 3:**
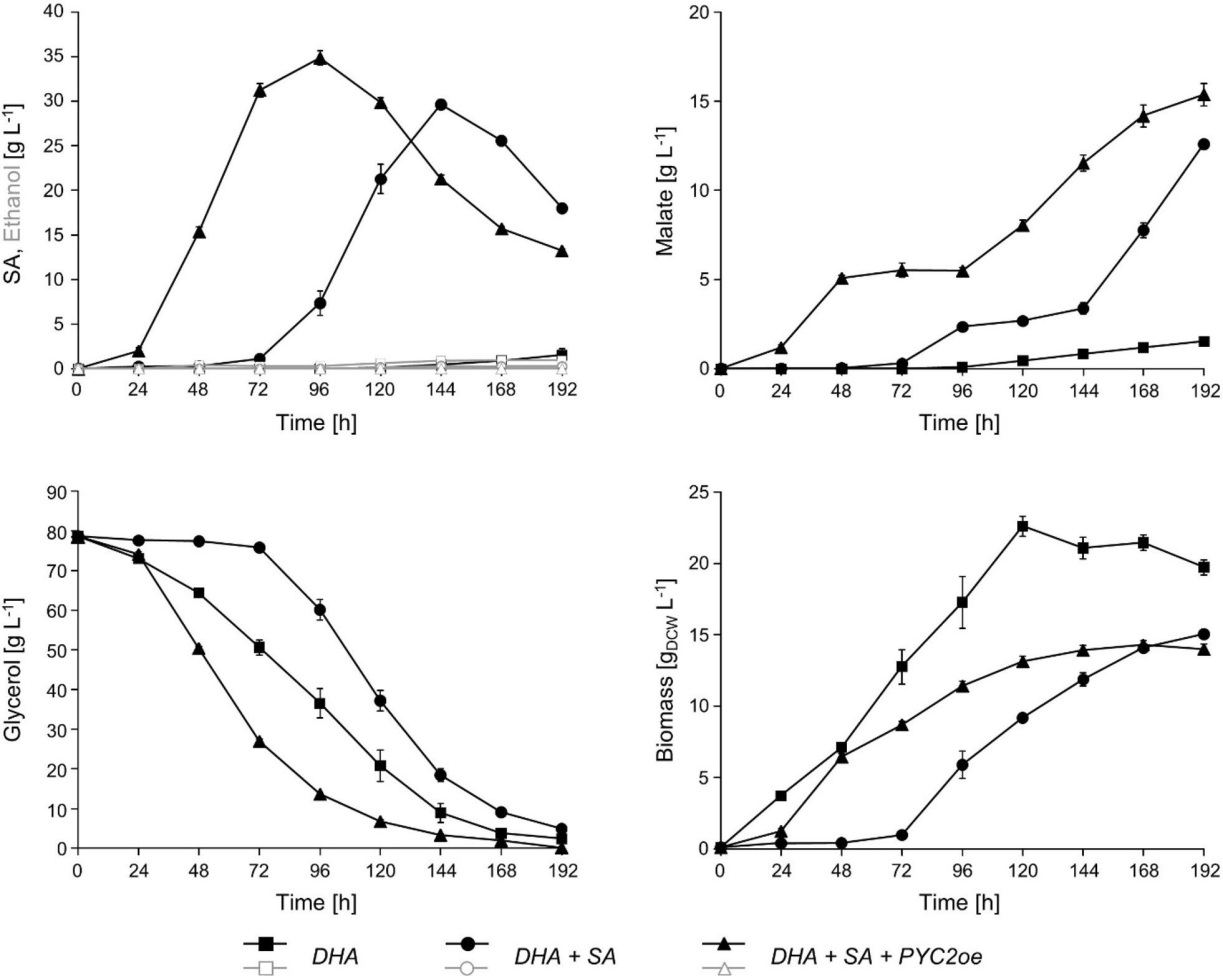
Performance of the 2nd-generation *S. cerevisiae* SA producers in shake flask cultivations supplemented with 30 g L^−1^ CaCO_3_. The new DHA pathway (baseline) strain was used as a control. The cultivations were performed in 500 mL shake flasks filled with 100 mL synthetic glycerol medium using urea as the nitrogen source. After addition of CaCO_3_ the cultures had a pH of ~ 6. HPLC analysis was used to determine the concentrations of glycerol, SA and malate in the culture supernatant. Biomass accumulation was measured by optical density at 600 nm (OD_600_). Notably, virtually no ethanol was formed during the cultivations and the pH remained relatively constant between pH 5 and 6 for the SA producing strains and between 6 and 7.5 for the control. Mean values and standard deviations were determined from three biological replicates. Strain abbreviations: *DHA* UBR2_CBS_-DHA(2), *DHA* + *SA* UBR2_CBS_-DHA-SA-AnDCT-02 (2), *DHA* + *SA* + *PYCoe* UBR2_CBS_-DHA-SA-AnDCT-02 (2)-PYC2oe

Next, we scrutinized the question whether the availability of bicarbonate, the co-substrate of the pyruvate carboxylase reaction, might have become rate-controlling in the strains with the optimized rTCA pathway. To test this hypothesis, we added CaCO_3_ to shake flask cultivations. It has previously been shown that the addition of CaCO_3_ to shake flask cultivations for organic acid production buffers the cultivation medium [22, 41, 42] and, at the same time, increases the bicarbonate concentration in the medium [41]. We tested the effect of CaCO_3_ addition to the culture medium for the strains UBR2_CBS_-DHA-SA-AnDCT-02 (2) and UBR2_CBS_-DHA-SA-AnDCT-02 (2)-PYC2_oe_ as well as for the control strain UBR2_CBS_-DHA (2) in synthetic glycerol medium (Fig. 3).

First, it should be noted that the presence of CaCO_3_ indeed showed a buffering effect as the determined pH of the respective cultures remained relatively constant. While the pH in the cultures of the two SA producing strains was between 5 and 6 throughout the cultivation, the pH of the culture containing the reference strain UBR2_CBS_-DHA (2) fluctuated between pH 6 and pH 7.5 (Additional file 1: Figure S4). In the first phase of the cultivation, both SA producing strains (with and without overexpression of *PYC2*) behaved similarly as compared to the experiment without CaCO_3_ addition. Particularly, the strain overexpressing *PYC2* rapidly accumulated SA in the medium. However, this time cell growth and SA production did not cease abruptly but continued until a maximum SA titer of ~ 35 g L^−1^ was reached after 96 h of cultivation (Fig. 3). The highest SA yield (0.6 g g^−1^ corresponding to 0.63 Cmol Cmol^−1^) was observed after 72 h. The presence of CaCO_3_ allowed all strains to consume significantly more glycerol. Virtually, no ethanol was formed (Fig. 3). Notably, significant amounts of malate (MA) were additionally secreted into the medium (Fig. 3). The MA yield after 72 h (i.e. when SA yield was highest) was about 0.1 Cmol Cmol^−1^. At this time point, 53.2% of the electrons that were available in the consumed glycerol ended up in dicarboxylic acids (SA plus MA). After this point in time, SA titers decreased while MA titers increased concomitantly, indicating SA uptake and partial conversion to MA.

### Characterization of strain UBR2_CBS_-DHA-SA-AnDCT-02 (2)-PYC2oe in pH-controlled bioreactor experiments with CO_2_-sparging and off-gas analysis

**Fig. 4:**
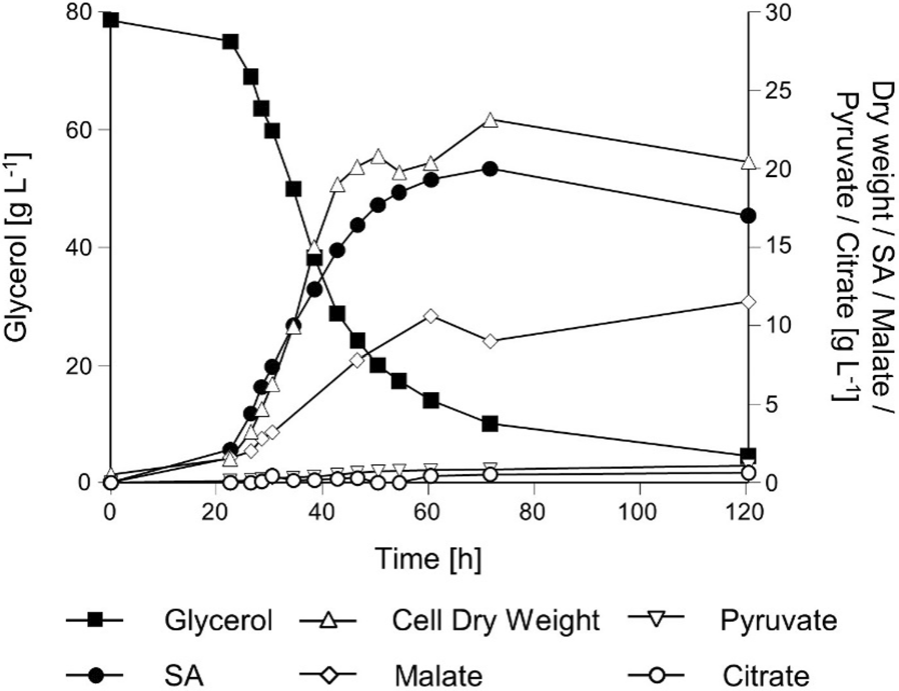
Physiological characterization of the *S. cerevisiae* strain UBR2_CBS_-DHA-SA-AnDCT-02 (2)-PYC2oe in batch cultivations in 2-L bioreactors. Synthetic medium containing 75.6 g L ^1^ glycerol as the carbon source and ammonium sulfate as nitrogen source (pH was kept at a level of 5.0 throughout the cultivation) was used. One representative out of two experiments is shown

Addition of CaCO_3_ to shake flask cultures could have improved strain performance by buffering the culture pH or increased availability of bicarbonate for the pyruvate-kinase reaction. To better control these culture parameters and to quantify CO_2_ production/utilization, we tested the strain UBR2_CBS_-DHA-SA-AnDCT-02 (2)-PYC2oe in controlled bioreactor cultures. As the previous strain UBR2_CBS_-DHA-SA-AnDCT-02 was also characterized in bioreactors [17], we used exactly the same experimental conditions in order to be able to compare the results. The synthetic medium contained ammonium sulfate as a nitrogen source and 75.6 g L^−1^ glycerol, the pH was maintained at a value of 5.0 and the cultures were sparged with CO_2_ enriched air (~ 10% (v/v) CO_2_) at a rate of 500 mL min^−1^. Under these conditions, a maximum SA titer of ~ 20 g L^−1^ was obtained with a yield of ca. 0.35 g g^−1^ glycerol consumed and a maximum specific SA production rate of 0.25 g g_DCW_^−1^ h^−1^ (Fig. 4). The strain also accumulated up to 9.0 g L^−1^ of malic acid (0.11 g g^−1^ glycerol consumed) under these conditions (Fig. 4), which was also observed in shake flask cultures with addition of CaCO_3_ (Fig. 3). The strain also produced small amounts of pyruvate and citrate in the bioreactor experiment, however, the concentrations of these organic acids (~ 1.1 and 0.6 g L^−1^, respectively) were significantly lower compared to the values obtained for the previously published SA producing strain under the same conditions [17].

**Fig. 5:**
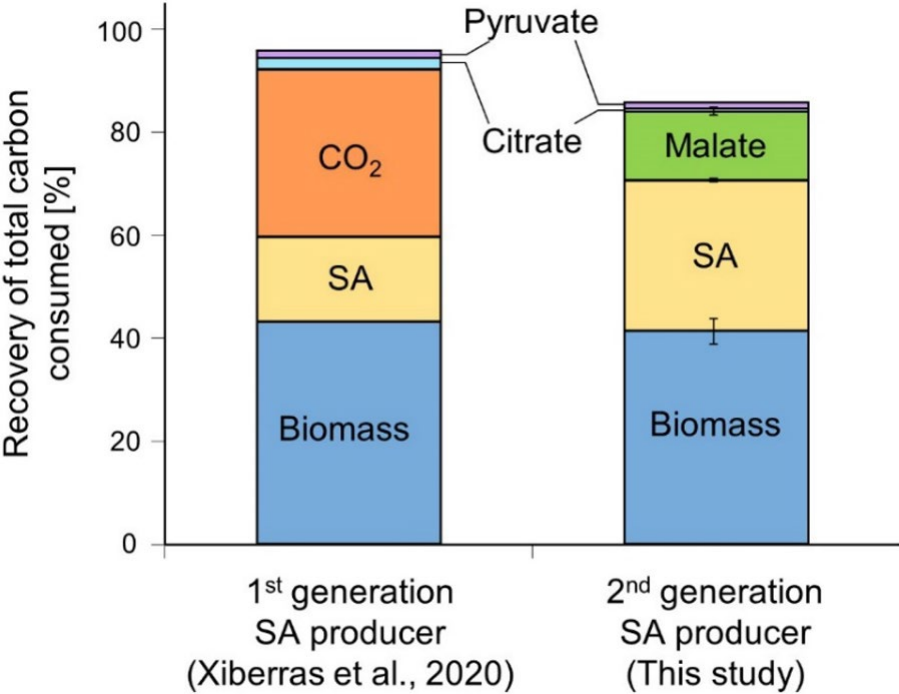
Fate of consumed carbon in the strain *DHA-SA* [17] and the 2nd-generation SA producer *DHA-SA-PYC* constructed in the current study. Data on the left is derived from Xiberras et al. [17], data on the right represents the fate of the consumed carbon in the productive phase (0–71.75 h) of batch bioreactor cultivation in the present study. Total carbon consumption was determined by adding the net consumption of CO_2_ and glycerol. Biomass and metabolite formation were determined by cell dry weight and HPLC analysis. Effects of dilution due to KOH addition for pH control and withdrawal of sample volume were taken into account. Data represents the average ± mean deviation of duplicate experiments and represents the carbon ending up in various products as a fraction of total carbon consumed. Due to technical difficulties, CO_2_-logging data of the first hour of one replicate was omitted. Strain abbreviations: *DHA* + *SA* UBR2_CBS_-DHA-SA-AnDCT-02, *DHA* + *SA* + *PYCoe* UBR2_CBS_-DHA-SA-AnDCT-02 (2)-PYC2oe

Based on the concentration of CO_2_ in the gas mixture used for sparging and continuous measurement of the bioreactor off-gas, we found that in the productive first 71.75 h of the cultivation, net consumption of CO_2_ occurred (Additional file 3). This is in clear contrast to the behavior of the previously published strain for which ~ 32.7% of the consumed carbon was found in formed CO_2_ at the corresponding time point (after 71.5 h of cultivation). To compare the distribution of consumed carbon in both strain designs, we therefore included the net consumption of CO_2_ (representing 5.7 ± 3.4% of the consumed carbon) for the new strain design (Fig. 5). Whereas a similar fraction of carbon ended up in the formation of biomass, the strain modifications in this study were sufficient to completely eliminate CO_2_ formation and resulted in a substantially higher yield of succinic acid, as well as significant by-product formation in the form of malic acid.

## Discussion

The application of *S. cerevisiae* as a robust fungal cell factory holds great promise for the fermentative production of organic acids at low pH. As explored in the introduction, the use of glycerol is highly attractive for achieving maximum CO_2_-fixation capacity and product yield. Here, we constructed a 2nd-generation strain producing SA from glycerol, mainly by applying strong glycerol-active promoters and increasing the activity of the endogenous *PYC2*. As a result, we provided a baker’s yeast strain able to produce SA from glycerol with a maximum yield of 0.60 g g^−1^ glycerol (achieved in shake flasks with addition of CaCO_3_ after 72 h of cultivation) corresponding to 0.62 Cmol Cmol^−1^. Although the changes in the genetic constitution of our 2nd generation strains compared to the 1st generation strain described by Xiberras et al. [17] regarded both the DHA module and the rTCA pathway, we have good reasons to believe that the latter modification was mainly responsible for the improved SA production. We combined the 2nd generation rTCA pathway (including SA export) and the 1st generation DHA pathway (including glycerol import) and obtained a maximum SA yield of 0.51 ± 0.04 g_SA_ g_glycerol_^−1^ in CaCO_3_ supplemented shake flask cultivations, a value directly comparable with the results presented for strain UBR2_CBS_-DHA-SA-AnDCT-02 (2) in this work. Apart from the remarkably increased product yield, there are two results of the current study which stand out compared to the 1st generation strain published by Xiberras et al. [17]. First, the abolishment of the glyoxylate cycle (by deletion of *ICL1*) did not result in a decrease in SA titer or yield. Second, the off-gas analysis revealed net CO_2_ consumption during the productive phase of the cultivation (Fig. 5). Taken together, the results presented in this work strongly support our hypothesis that the carbon flux into the rTCA pathway in our new strains is significantly higher compared to our 1st-generation strain.

The results obtained in the current study have resulted from shake flask cultivations and bioreactor experiments without particular efforts to achieve competitive titer, yield and productivity. Therefore, there is still a lot of room for improvement for these measures for SA production from glycerol with our strain. Notably, the knowledge obtained in previous approaches to increase SA yield and titer from glucose of an SA-overproducing industrial *S. cerevisiae* strain by bioprocess engineering can easily be applied to our strain and glycerol as the source of carbon. For example, Wahl et al. [32] used nitrogen limitation in fed-batch process while Liu et al. [43] applied near-zero growth conditions (retentostat).

In this work, we focused on the production of SA from glycerol, as the use of this carbon source allows for increased CO_2_ fixation compared to sugars [2]. Although the strain designs in this work already achieved relatively high product yields, industrial implementation also requires high titers and production rates. The production rate obtained in our bioreactor experiment (0.25 g g_DCW_^−1^ h^−1^) is considerably lower than published values obtained on sugars using industrial strains producing SA from glucose [43]. In this context, one has to consider that the flux through glycolysis in the Crabtree-positive yeast *S. cerevisiae* is extraordinarily high on glucose [44]. It will be a future challenge to achieve similarly high fluxes on glycerol. It has been a promising observation of the current study that the specific glycerol consumption rate (readout for glycolytic flux) was already strongly enhanced by the new SA module when compared to either the baseline strain (Additional file 1: Fig. S4) or the strain constructed by Xiberras et al. [17]. The overexpression of *PYC2* further increased this measure (Additional file 1: Fig. S3). The simplest explanation for this effect is that a more efficient rTCA pathway established a redox-factor (NADH) neutral (fermentative) route from the substrate to the product (Fig. 1). Therefore, any glycerol converted via this (rTCA) pathway is not dependent on subsequent NADH reoxidation via respiration. In fact, respiratory capacity could have been a bottleneck in the previous strain design.

The data of this study show that our best strain accumulated malate, an intermediate of the rTCA SA production pathway, in the bioreactor cultivations (in the presence of increased CO_2_ concentrations in the gas phase) and in the shake flask experiments conducted in the presence of CaCO_3_. In contrast to ordinary shake flask cultures, both cultivation procedures directly or indirectly controlled the pH and increased the level of bicarbonate. An increased concentration of the latter is supposed to thermodynamically favor the flux from pyruvate to oxaloacetate. Notably, other authors also observed malate accumulation in the above-mentioned strain engineered by the company DSM for SA production from glucose and also equipped with the rTCA pathway including *PYC2* overexpression [32]. In the respective study, CO_2_ concentration was also increased during the bioreactor experiments. Taken together, our results support the hypothesis of the latter authors that fumarase seems to become rate-controlling in strains equipped with the rTCA pathway. In general, one should also consider that the *An*DCT-02 transporter may possess malate export activity like its close homologues [45–47], thus supporting malate secretion under conditions which lead to malate accumulation. Further pathway optimization as well as utilization of an exporter which lacks the affinity for malic acid may ensure that even more substrate gets converted into SA, at the expense of MA titers and yields.

In general, a ^13^C-metabolic flux analysis would be very valuable to guide further metabolic engineering steps for SA production from glycerol. However, the precise analysis of intracellular fluxes requires compartment-specific metabolome analysis for which a method was previously developed in mammalian cells [48], but still needs to be established for yeast. Moreover, a reliable metabolic model adapted to glycerol as the carbon source (including a corrected biomass equation) is crucial and currently under preparation.

## Conclusion

In conclusion, we provide here a highly promising *S. cerevisiae* strain for the production of SA from glycerol. Solely considering the redox balance, the envisaged pathway from glycerol to SA has the potential to achieve yields close to the theoretical maximum similar to ethanol fermentation from glucose. However, to achieve these yields, the overall ATP stoichiometry and its impact on flux distributions has also to be taken into account and the production of an organic acid is more challenging in this regard compared to ethanol as elaborated by de Kok et al. [49]. As a consequence, in the strain designs described in this work, some of the glycerol needs to be respired which imposes a limit on the maximum yield achievable with our current strain. Therefore, future work focused on the energetic requirements of SA formation from glycerol and SA export could be a valuable step towards even better strain designs.

## Supplementary Information

**Additional file 1: Figure S1. **History of constructing the 2nd-generation SA producers. **Figure S3.** **Figure S2. **Comparison of the DHA pathway (baseline) strains used in the study of Xiberras et al. [17] and in the current work. Specific and volumetric glycerol consumption rates of 2nd-generation SA producers in ordinary shake flask cultures. **Figure S4.** Time courses of medium pH for the shake flask cultivations supplemented with 30 g L^−1^ CaCO_3_.

**Additional file 2:** **Table S1.** Plasmids used in this study. **Table S2.** Primers used for plasmid construction. **Table S3.** Primers and templates used for amplification of disruption and integration cassettes. **Table S4.** Strains used in this study. **Table S5.** Genotypic comparison of SA strains published in Xiberras et al. [17] and those used in this study. **Table S6.** Coding sequences for heterologous genes in 2nd generation SA module plus transporter.

**Additional file 3.** Raw online data, offline (sampling) data and derived calculations of bioreactor experiments with strain DHA-SA-PYC.

## Data Availability

All data generated or analyzed during this study are included in this published article [and the additional files].

## Abbreviations

Cmol: Carbon mole
DHA: Dihydroxyacetone
d.o.r: Degree of reduction
HPLC: High performance liquid chromatography
l-G3P: l-glycerol-3-phosphate
MA: Malic acid
PYC2oe: *PYC2* Overexpression
RI: Refractive index
rTCA: Reverse tricarboxylic acid cycle
SA: Succinic acid
