# Supplementary material for: Carbon dioxide fixation via production of succinic acid from glycerol in engineered Saccharomyces cerevisiae
Source: Microb Cell Fact. 2022 May 28;21:102. doi: 10.1186/s12934-022-01817-1 (PMC9148483; doi:10.1186/s12934-022-01817-1)
Supplement: Supplementary file 1 — Additional file 1: Figure S1. History of constructing the 2nd-generation SA producers. Figure S3. Figure S2. Comparison of the DHA pathway (baseline) strains used in the study of Xiberras et al. [17] and in the current work. Specific and volumetric glycerol consumption rates of 2nd-generation SA producers in ordinary shake flask cultures. Figure S4. Time courses of medium pH for the shake flask cultivations supplemented with 30 g L−1 CaCO3. [file 12934_2022_1817_MOESM1_ESM.docx]

Malubhoy et al.

**Carbon dioxide fixation via production of succinic acid from glycerol in engineered *Saccharomyces cerevisiae***

**Additional File 1: Supplementary Figures**

**
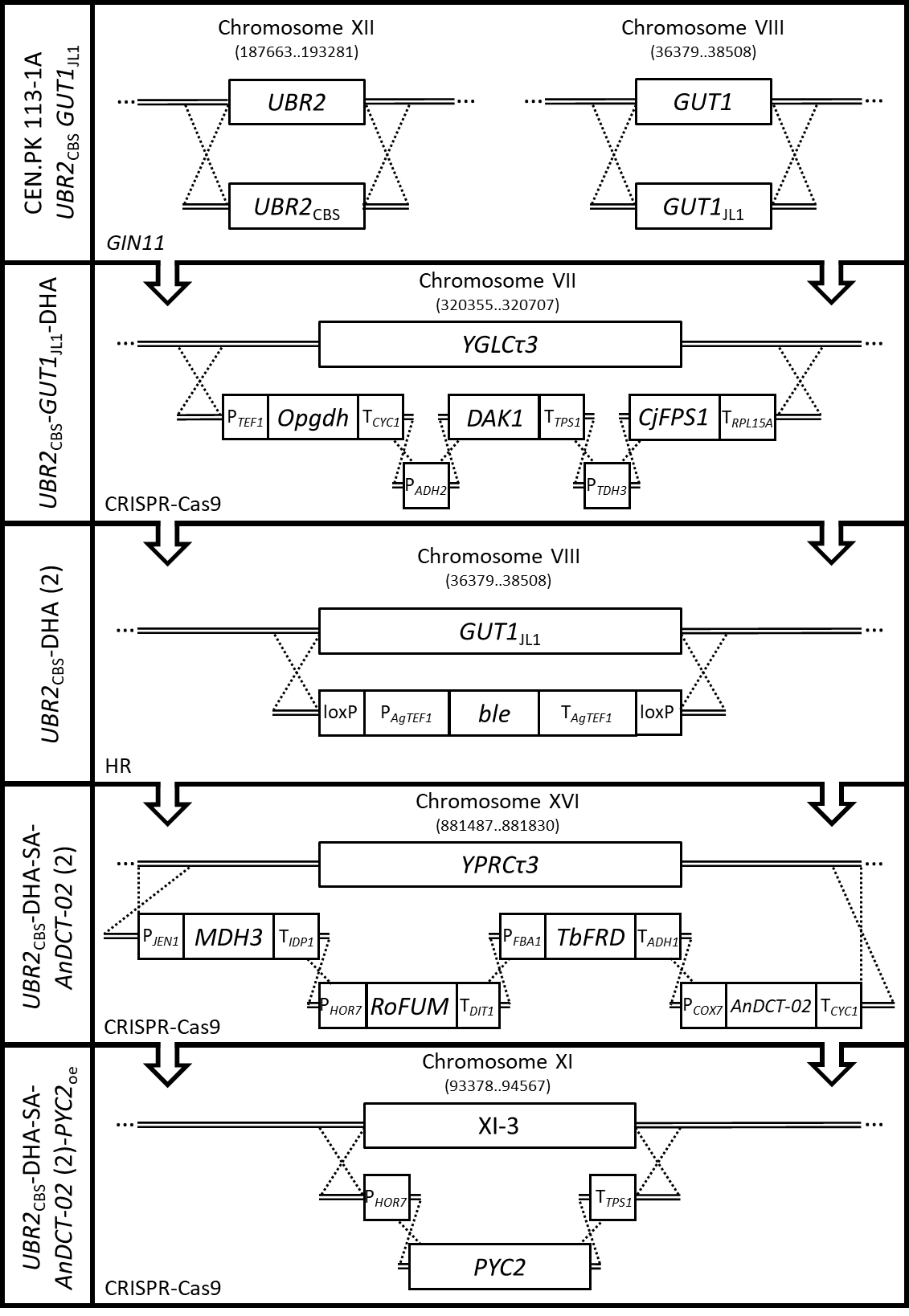
**

**Figure S1. History of constructing the 2^nd^-generation SA producers.** The strain CEN.PK 113-1A *UBR2*_CBS_ *GUT1*_JL1_ shown at the top was used as a starting point to construct all other strains used in this study and has been previously described by Ho et al (2018). The strain *UBR2*_CBS_-DHA-SA-*AnDCT-02*-*PYC2*_oe_, shown at the bottom, was constructed stepwise in four steps: 1) the expression cassettes for *Ogataea parapolymorpha*’s glycerol dehydrogenase (*Opgdh*), the endogenous dihydroxyacetone kinase (*DAK1*), and *Cyberlindnera jadinii*’s aquaglyceroporin (*CjFPS1*) were integrated into the *YGLCτ3* genomic locus using the CRISPR/Cas9 technology; 2) the *GUT1* gene was disrupted with an expression cassette containing the phleomycin resistance gene from *Streptoalloteichus hindustanus* (*Shble*) and flanked with loxP sites via homologous recombination (HR); 3) the expression cassettes for the endogenous, retargeted malate dehydrogenase (*MDH3*), *Rhizopus oryzae*’s fumarase (*RoFUM*), *Trypanosoma brucei*’s retargeted fumarate reductase (*TbFRD*), and *Aspergillus niger*’s dicarboxylic acid transporter (*AnDCT-02*) were integrated into the *YPRCτ3* genomic locus using the CRISPR/Cas9 technology; 4) the expression cassette for the endogenous pyruvate carboxylase overexpression (*PYC2*) was integrated into the XI-3 genomic locus using the CRISPR/Cas9 technology. The genomic loci coordinates are relative to the reference strain *S. cerevisiae* S288C’s genome in the *Saccharomyces* Genome Database (SGD). The integration sites *YGLCτ3* and *YPRCτ3* have been described by Flagfeldt et al. (2009) and XI-3 has been described by Jessop-Fabre et al. (2016).


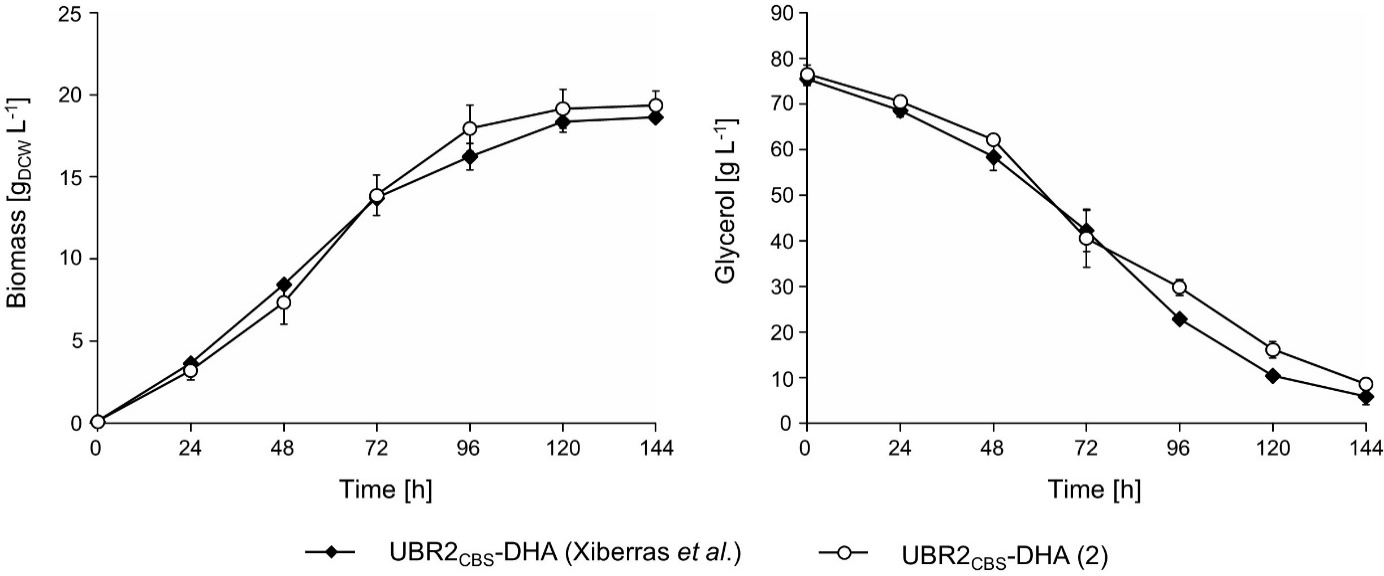


**Figure S2. Comparison of the DHA pathway (baseline) strains used in the study of Xiberras et al. (2020) and in the current work.** In both strains, the native *UBR2* allele was swapped by the respective allele from CBS 6412-13A as described by Swinnen et al. (2016). In the strain used by Xiberras et al. (2020), the DHA pathway module consists of expression cassettes for *O. parapolymorpha* glycerol dehydrogenase (*Opgdh*), the *C. jadinii* aquaglyceroporin encoded by *FPS1* (*CjFPS1*) and two cassettes for overexpression of endogenous dihydroxyacetone kinase (*DAK1*) under the control of different promoters and terminators. The four expression cassettes were integrated into the *GUT1* locus abolishing the activity if glycerol kinase (the first enzyme of the endogenous L-G3P pathway for glycerol catabolism). In the strain constructed for the current study, the DHA pathway module consists of expression cassettes for *Opgdh*, *CjFPS1* and a single cassette for overexpression of *DAK1* under the control of promoter exhibiting strong activity in medium containing glycerol as the carbon source. The cultivation was performed in 500 mL shake flasks filled with 100 mL synthetic glycerol medium at pH 4 using urea as the nitrogen source. HPLC analysis was used to determine the concentrations of glycerol. Biomass accumulation was measured by optical density at 600 nm (OD_600_). Mean values and mean deviations from at least two biological replicates are shown.


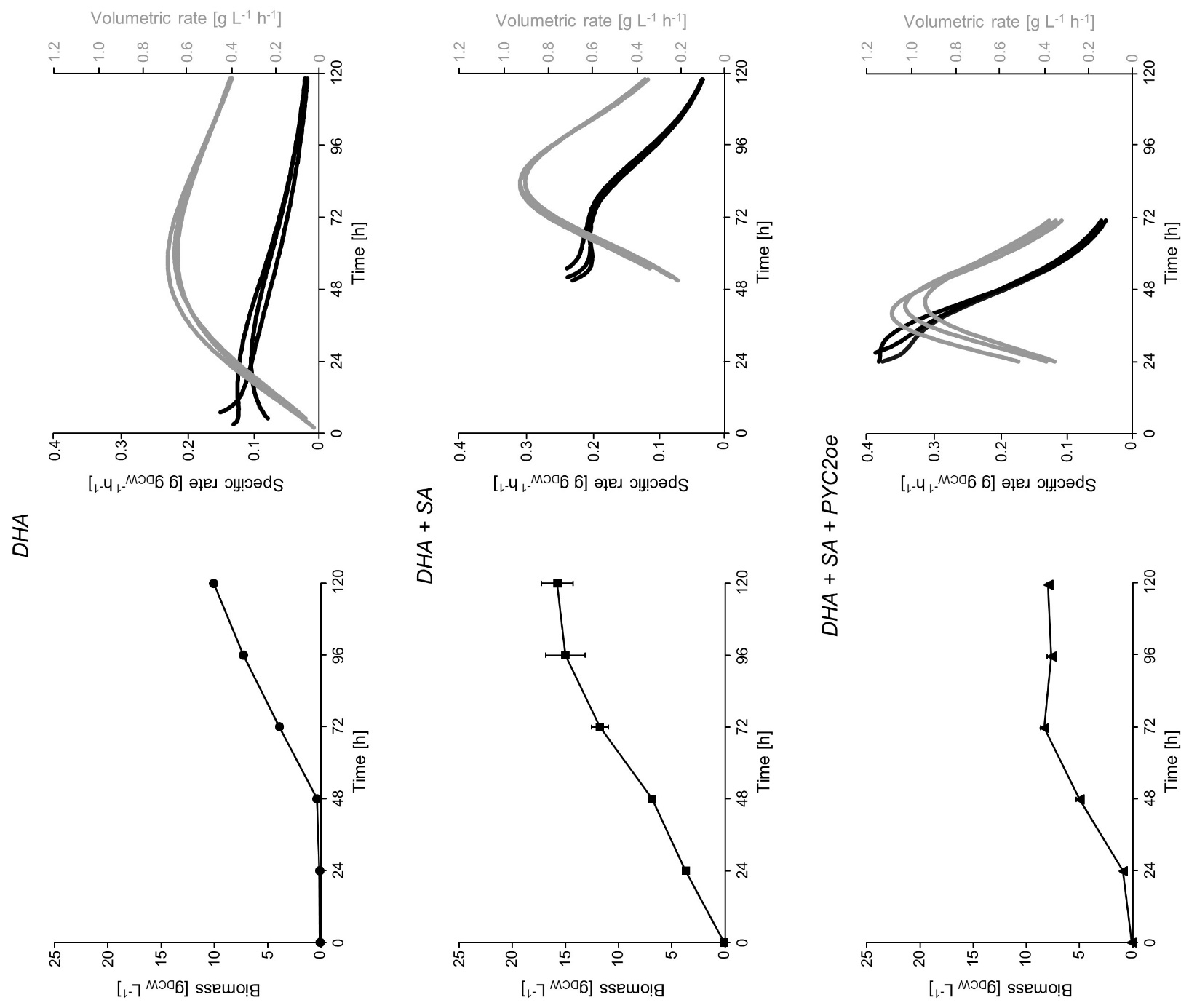


**Figure S3.** **Specific and volumetric glycerol consumption rates of 2^nd^-generation SA producers in ordinary shake flask cultures.** The graphs on the left show the average biomass accumulation for each strain in the respective time period. The rate calculations are based on the data shown in Fig. 2. Values for optical densities were converted into dry cell weight using a standard curve as described in Materials and Methods. The graphs on the right show the respectively calculated volumetric (grey) and specific rates of glycerol consumption (black) for three biological replicates of the respective strain. Strain abbreviations: *DHA*: UBR2_CBS_-DHA (2); *DHA+SA*:UBR2_CBS_-DHA-SA-AnDCT-02 (2); *DHA+SA+PYCoe*:UBR2_CBS_-DHA-SA-AnDCT-02 (2)-PYC2oe.


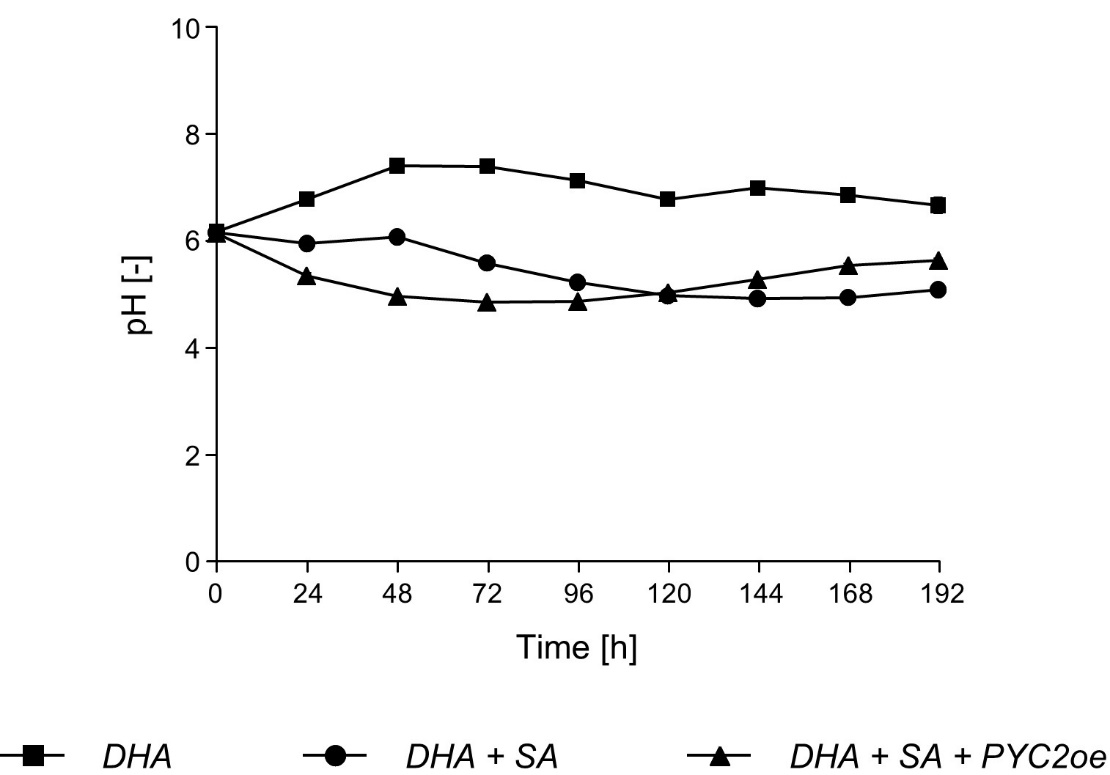


**Figure S4.** **Time courses of medium pH for the shake flask cultivations supplemented with 30 g L^-1^ CaCO_3_** This Figure corresponds to the experimental data shown in Fig. 3 of the main manuscript.
